# The complete mitochondrial genome of Japanese sea lion, *Zalophus japonicus* (Carnivora: Otariidae) analyzed using the excavated skeletal remains from Ulleungdo, South Korea

**DOI:** 10.1080/23802359.2021.1945503

**Published:** 2021-10-14

**Authors:** Eun-Bi Kim, Myung Joon Kim, InSeo Hwang, Hye-Min Park, Sang Heon Lee, Hyun-Woo Kim

**Affiliations:** a Industry 4.0 Convergence Bionics Engineering, Pukyong National University, Busan, Republic of Korea; b Department of Oceanography, Pusan National University, Busan, Republic of Korea; c Marine Ecosystem Management Team, Korea Marine Environment Management Corporation, Seoul, Republic of Korea; d Department of Marine Biology, Pukyong National University, Busan, Republic of Korea

**Keywords:** *Zalophus japonicus*, mitogenome, phylogeny, pinnipeds, Korea

## Abstract

The Japanese sea lion, *Zalophus japonicus*, is an extinct pinniped species, which had inhabited along the coast of the Japanese archipelago and Korean peninsula. Its mitochondrial genome was determined by the assembly of PCR amplicons from the skeletal remains excavated from the Ulleungdo, South Korea. The whole mitogenome was 16,698 bp in length, which encoded 13 protein-coding genes (PCGs), 22 transfer RNAs (tRNA), 2 ribosomal RNAs (rRNA), an origin of light-strand replication (OL), and a control region (D-loop). Unusual start codons were identified in ND2 (ATA), ND3 (ATA), and ND5 (ATC), while COIII, ND3, and ND4 were terminated with an incomplete stop codon (T–/TA-). Phylogenetic analysis showed that *Z*. *japonicus* was a sister species to *Z*. *californianus* with 98.61% nucleotide sequence identity among 11 pinniped species in the infraorder Pinnipedia, which supported the previous results. The complete mitochondrial genome sequence of *Z*. *japonicus* would be valuable information for its restoration and the evolutional understandings of pinniped species in the Pacific Ocean.

The Japanese sea lion, *Zalophus japonicus* is one of three species in the genus *Zalophus* according to the World Register of Marine Species (WoRMS). The DNA analysis suggested that this species diverged from its relative *Z. californianus* in the early Pleistocene approximately 2 million years ago (Sakahira and Niimi 2007). *Zalophus japonicus* inhabited along the northwest Pacific coastline ranging from Japan and Russia (Kamchatka and Sakhalin) to Korea, and in particular, the Ulleungdo and Dokdo have been known as one of the main Korean habitats (Rice 1998).

The population of *Z. japonicus* declined drastically in the early twentieth century due to overhunting and the species was consequently classified as extinct by the International Union for Conservation of Nature (IUCN) in 1994 (Lowry 2017). Several countries including Korea, Japan, and Russia have collaborated to restore the pinniped resources along the northwest Pacific region but the genetic information of *Z*. *japonicus* is still limited. We here first reported the complete mitochondrial genome of *Z*. *japonicus* obtained from the excavated skeletal remains and analyzed its phylogenetic relationship with the other pinniped species.

The several bones of *Z. japonicus* were collected from the Ulleungdo (37°30′56.9″N, 130°47′38.3″E) during a research survey funded by the Ministry of Oceans and Fisheries, Korea. Genomic DNA was extracted from bone marrow using DNeasy^Ⓡ^ Blood & Tissue Kit (QIAGEN, Hilden, Germany) according to the manufacturer’s protocol with a slight modification. The mitochondrial COI region of the skeletal sample showed the highest sequence identity to *Z*. *californianus* (98.31%, GenBank number: AM181017), followed by *Eumetopias jubatus* (94.94%, GenBank number: GU475464). Since only the sequences for the D-loop control region of *Z. japonicus* were available in the GenBank database, species identification of the skeletal sample was reconfirmed by the comparison of the control region. Amplified its control region showed 99.39% and 96.57% identity to *Z*. *japonicus* from the Aichi Prefecture in Japan (GenBank number: AB262362) and from the Dokdo of Korea (GenBank number: MK360815), respectively (Sakahira and Niimi 2007; Lee et al. 2019). The identified skeletal remains were stored at the National Marine Biodiversity Institute of Korea (https://www.mabik.re.kr/html/en/, Hyuck Joon Kwun, kwunhj@mabik.re.kr) under the number MABIK MM00000008.

A total of 16 PCR fragments were amplified by long-PCR strategy with the degenerated primers, which were designed by currently reported pinniped mitogenome sequences in the GenBank database. Each long PCR fragment was determined by Sanger sequencing. The whole mitochondrial genome of *Z*. *japonicus* was constructed by assembling PCR fragments using Geneious software (Kearse et al. 2012) with the mitogenome sequence of *Z*. *californianus* (GenBank number: AM181017) as a reference. Secondary structures of tRNAs were predicted using tRNAScan-SE online program (Lowe and Chan 2016). The phylogenetic tree was constructed with the maximum likelihood algorithm under the 1000 replication bootstrap by MEGA version 7 (Kumar et al. 2016).

The circular complete mitochondrial genome of *Z*. *japonicus* was 16,698 bp in length (GenBank number: MW563321). It has consisted of 13 protein-coding genes (PCGs), 22 transfer RNAs (tRNA), 2 ribosomal RNAs (rRNA), an origin of light-strand replication (OL), and a non-coding control region (D-loop), which showed typical mitochondrial genome feature. The overall nucleotide composition was 33.5% A, 26.8% C, 13.8% G, and 25.9% T, respectively. Among 37 genes, 28 genes (12 PCGs, 14 tRNA, and 2 rRNAs) were encoded on heavy (H) strand, and remaining 9 genes (ND6 and eight tRNA) were located on light (L) strand. Three PCGs, *ND2*, *ND3*, and *ND5*, were initiated with an unusual start codon (ATA, ATC), while incomplete stop codons (T––/TA–) were identified in three PCGs including *COIII*, *ND3*, and *ND4*. All tRNA genes were predicted to form a typical clover-leaf structure except for tRNA^Ser-GCT^ without D-arm. The phylogenetic analysis of *Z*. *japonicus* was conducted with its relatives in the infraorder Pinnipedia using concatenated sequences of 13 PCGs (Figure 1).

**Figure 1.**
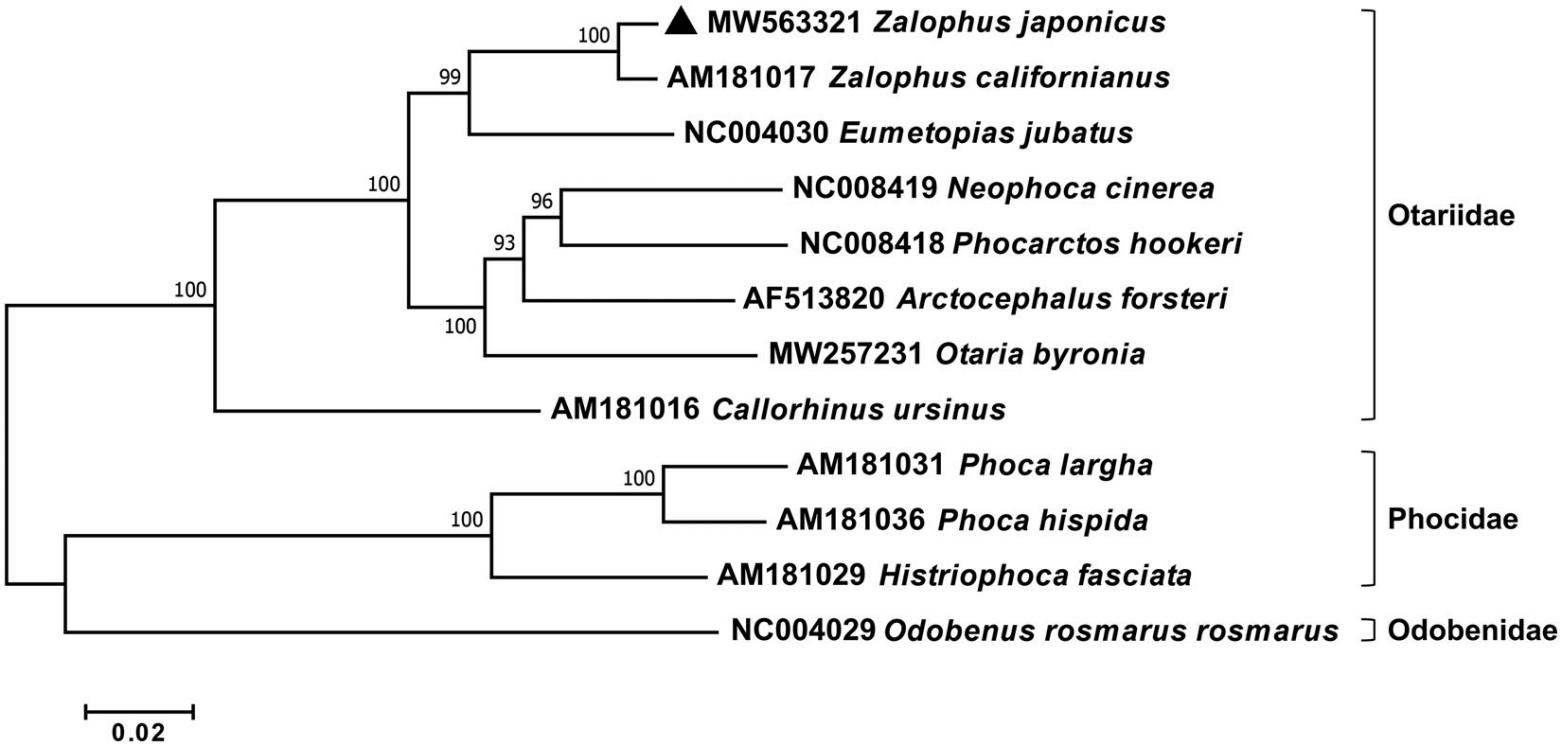
Phylogenetic relationship of *Z. japonicus* with other 11 pinnipeds in the infraorder Pinnipedia. A phylogenetic tree was constructed with 13 mitochondrial protein-coding genes using the maximum-likelihood algorithm by MEGA version 7 software. The bootstrap values generated from 1000 replicates are shown at each branch and a black triangle represents *Z. japonicus* as determined in this study.

The phylogenetic tree showed that two species in the genus *Zalophus* formed a separate clade, which is distinct from those in the family Otariidae. *Z*. *japonicus* was most closely related to *Z*. *californianus* with 98.61% sequence identity, followed by *E*. *jubatus* with 93.40% identity. This is the first report of the complete mitochondrial genome sequence of *Z*. *japonicus*, which would provide a valuable genetic information for its restoration as well as for the evolutionary relationships of pinniped species in the Pacific Ocean.

## Data Availability

The genome sequence data that support the findings of this study are openly available in GenBank of NCBI at https://www.ncbi.nlm.nih.gov/ under the accession no. MW563321. The associated BioProject, SRA, and Bio-Sample numbers are PRJNA714485, SRR13962179, and SAMN18310374 respectively.
